# Bibliography

**Published:** 1893-02

**Authors:** 


### Bibliography.

---

Methods of Filling Teeth. By Rodrigues Ottolengui, M.D.S. Two Hundred Pages, with Two Hundred and Thirty-six Illustrations. S. S. White Dental Manufacturing Company, Philadelphia. Claudius Ash & Sons, Limited. London, 1892.

The question may be asked, Why another book on the methods of filling teeth, when the processes which give the best results have been described over and over again? The answer may, perhaps, be found in the fact that new generations of professional workers are coming on the stage yearly, and it may be well from time to time to give them fresh thought on a somewhat threadbare subject.

The fact must be recognized that it is not always the manipulative skill displayed in a book of this character that is most needed. The influence for a higher ability manifested in its pages and the preservation of procedures which have built up the practice of dentistry in the past will be its greatest value. There has always been a danger that the skill acquired with so much effort in former periods might be lost in the conflict between self-interest on the one hand and indifferent teaching on the other. Hence it must be regarded as by no means waste energy to again present this subject of filling teeth in book form.

The contents have in a measure become familiar to readers of the *Dental Cosmos* through their serial publication in that journal. This plan of introducing a book is certainly not to be commended. While it may give general notoriety, it works ultimately to the injury of the book and places the reader at a disadvantage, for those who peruse the articles as they appear secure but a limited idea of the scope of the work, and may never take the trouble to re-read it. The result is frequently adverse and undeserved criticism.

A connected and careful reading by the reviewer leads to the opinion that in attractive style and thoroughness of description it has no equal in the publications upon this special subject,—that of filling with cohesive gold.

It must be remembered, in judging of this book, that the author makes no pretence to explaining other modes; indeed, he frankly acknowledges that he "has never used non-cohesive gold," therefore criticism is disarmed in this direction. He also states that he has "no broad claims to originality in connection with the methods described."

The style of the book, while attractive, may be an injury to it as a text-book, as there is a certain amount of delving to be done through paragraphs to seek the practical matter important to the beginner.

The author has started out with the idea of exhibiting the modes of doing a certain thing in the best manner possible, and in doing this he has certainly ignored conditions. He makes no account of age, character of tooth-tissue, systemic conditions, temperament of individuals, and the hundred and one things that prove such important factors in the success or failure of an operation.

While this may be well from his stand-point, it is a fatal error when considered from that of the partially-trained man. It is a return, in a measure, to that happily exploded idea, "That whatever is worth filling at all is worth filling with gold."

When the filling of teeth properly seems to be relegated, as at the present time, to enthusiasts, and regarded by the practical money-maker as waste time, it is a satisfaction to find one capable of describing how this should be done to produce the best results without regard to the financial rewards involved.

In considering this book in detail there will be found much to criticise.

It would be interesting to know where the author gets his authority for the assertion that "lead has been used in children's teeth, and occasionly in well-defined cup-shaped cavities in adult mouths." The writer of this review has been fairly conversant with all forms of filling-material, but he has yet to see lead-foil or to know of a case where it has been used. While he would not have it inferred that the author has not seen it, it would have added much to the assertion could he have given the authority for the statement.

The next paragraph, on "Tin," is a surprise, and it must be regretted that the author's prejudices against this material allowed him to make an *absolutely erroneous statement.* He writes, "It has also been made into foil and used as soft or non-cohesive fillings are made. Before the introduction of amalgam, it occupied the same place which that material too often does now,—for the filling of teeth *where a high fee could not be charged.* Except for this purpose, *which is an unprofessional one*, it has little value." No one who has used tin, as it should be used, and upon the same principles, so ably laid down by the author on cohesive gold, could so utterly condemn one of the most valuable materials ever placed in the hands of the dentist. No material has ever been more maligned than this, and it is incomprehensible how the author could have allowed himself to so distort the facts in regard to it.

His fling at tin and gold combined is equally unjust, and leads one to fear that his professional walk has been in narrow ways and that a blind adoration for gold has led him to overlook more humble but none the less valuable things. This is not the place to present arguments in favor of these two forms of fillings, but the author may be commended to seek the wisdom that comes by experience before he allows a second edition to contain such a radical error.

As an illustration of the weakness of his reasoning, a quotation is given in regard to the therapeutic action of oxychloride of zinc. " That an oxychloride filling does possess some therapeutic qualities seems indicated by the fact that it may usually be depended upon to lessen the sensitiveness of dentine if left in the cavity a month or more. This, of course, may occur under one of oxyphosphate simply because the tooth itself has altered, but as it has in my observation occurred more frequently with oxychloride, I deem it safe to say that an oxychloride filling has an obtunding effect." It is impossible to believe that the author does not know the rationale of the action of oxychloride in sensitive dentine, and why not say so instead of leaving the whole action involved in doubt?

The argument on page 63, in regard to the relative value of cohesive and non-cohesive gold, is decidedly lacking in force, and will not appeal to the thinking, practical minds who have had experience in all forms of this material. More than this, it seems wrong to mislead young men by such broad assertions. If his argument had been confined to proving that it is better to teach young men *one form of filling*, and that to be confined to cohesive in the first years of college training, no exceptions could be taken by the writer, but to ask the question, " Is there any advantage in non-cohesive gold . . . sufficiently important . . . to deserve a place in office practice?" and then to answer it by asserting, " If there is, I am ignorant of it," shows such a want of true knowledge as to almost throw a doubt over the conclusions arrived at as to the value of cohesive gold.

His argument that a student should be taught to use non-cohesive gold, in order that he may acquire " manual dexterity, which will quickly make him expert in the use of cohesive gold," is, in the opinion of the reviewer, fallacious in the extreme. The entire process of using non-cohesive differs so radically from that of cohesive gold, that he who learns the use of the former will have the greatest difficulty in properly acquiring the latter. The same rule applies to some extent to the use of the cohesive gold, but it has not the same deterring influence.

The author's argument against the V-shaped space, on page 85, will appeal to the contourist, who can see only in one direction, but to the men who lived in the days when these spaces were universal, the assertion that they " induced pyorrhœa" will be received with smiles of incredulity. It was not until long after the introduction of cohesive gold and the necessary adjuncts of rubber dam, ligatures, clamps, separators, etc., that we began to hear much of pyorrhœa alveolaris. The older dentists were not familiar with it; indeed, some went so far as to assert that such a pathological condition had no existence, as they had never met with it. The V-shaped spaces did not produce pyorrhœa, nor will the contour filling entirely prevent its development. The author must delve deeper to find a reason for his pathology.

The author very properly magnifies the danger of making "retentions" too near the pulp, but seems to be guilty of inconsistency in dilating upon the value of screws as retainers. The idea entertained by some, and implied by the author, that a drill hole, as a retaining-point made in dentine, involves danger to the pulp, has in the opinion of the reviewer but a small modicum of fact to support it. If these be made with a proper comprehension of the anatomy of the tissue, there is really less danger than from the filling itself, which is more often in closer proximity to the pulp than the artificial opening made could possibly be. There has probably been no one thing in operative dentistry more absurdly talked about than this, and we are glad to notice that our author does not bend entirely to the views held, but can see great virtue in a screw.

The pages from 104 to 170 seem too much of a repetition; certainly the good to be found in them hardly warrants restatement of methods practically described elsewhere.

More space has been given to a notice of this book than is usual to one of its size, but its importance is not to be calculated by pages. While it has been found necessary to call attention to a few blemishes in an otherwise admirable work, it must not be understood that the book as a whole is undervalued. There are books familiar to all readers that indicate epochs of thought and action. The world has recognized these and, while they may not have been welcomed, their influence has been acknowledged. It is with something of this view that the writer has risen from a critical reading of this. The courage which has led one of the younger men to plant his feet firmly upon the best, and only the best, work is not only in itself a cause of congratulation, but deserves something more, for it is a challenge, from a better standard of thought, to those who have made the filling of teeth almost a lost art. It means that the indifferent work so unpleasantly prominent in the last decade must stop and the profession return, if not to cohesive gold, to methods more in harmony with the skill it took many decades to develop.

Hence, while the criticism has been to exhibit some weak places, it must be apparent that no better book could be in the hands of both young and old. To the former it will be instructive, and to the latter an inspiration.

Materia Medica and Therapeutics. A Manual for Students and Practitioners. By L. F. Warner, M.D. Edited by Bern. B. Gallaudet, M.D. Lea Brothers & Co., Philadelphia.

Histology, Pathology, and Bacteriology. By Bennett S. Beach, M.D. Lea Brothers & Co., Philadelphia.

These two publications belong to the "Students' Quiz Series," but, differing from many of similar character, they have an intrinsic value peculiarly their own. That knowledge can be condensed so as to be made valuable as a means of study requires no argument; but to accomplish this satisfactorily requires talent not possessed by all who attempt it.

This applies especially to the manual on materia medica. The latter subject seems to possess special difficulties for a certain class of students. This is probably due to the mass of material found in the large works confusing to the untrained student, who finds it almost impossible to reduce it to proportions available for study, and, as he must necessarily depend on lectures and books, anything which will teach him how to study is of special value. That this manual accomplishes this must be acknowledged.

The same remark applies to the manual of "Histology, Pathology, and Bacteriology," but these subjects have an interest in themselves not to be found in a manual; but in the same direction of leading the mind directly to the main facts, it possesses a value equally with the preceding book.

